# Molecular Detection of Torque Teno Sus Virus and Coinfection with African Swine Fever Virus in Blood Samples of Pigs from Some Slaughterhouses in Nigeria

**DOI:** 10.1155/2016/6341015

**Published:** 2016-10-19

**Authors:** Pam D. Luka, Joseph Erume, Bitrus Yakubu, Olajide A. Owolodun, David Shamaki, Frank N. Mwiine

**Affiliations:** ^1^Department of Biomolecular Resources and Biolab Sciences, College of Veterinary Medicine, Animal Resources and Biosecurity, Makerere University, Kampala, Uganda; ^2^Biotechnology Division, National Veterinary Research Institute, Vom, Plateau State, Nigeria; ^3^Virology Division, National Veterinary Research Institute, Vom, Plateau State, Nigeria

## Abstract

Torque teno sus virus 1 (TTSuV1a/TTSuV1b) infection is present in pig herds worldwide. This study investigated the prevalence of TTSuV1a/TTSuV1b infections in domestic pigs from some slaughterhouses in Nigeria as well as coinfection with African swine fever virus (ASFV) and described the phylogeny in relation to global strains. One hundred and eighty-one (181) blood samples from four slaughterhouses were used for the study and viral nucleic acid detection was carried out by PCR. Comparative sequence analysis was carried out to infer phylogeny. The overall prevalence of TTSuV1a/b was 17.7%. Prevalence of individual genotypes was 10.5% and 7.2% for TTSuV1a and TTSuV1b, respectively. Coinfection of ASFV/TTSuV1a/b was 7.7% while that of TTSuV1a and TTSuV1b was 1.7%. ASFV alone was detected in 11.91% of the total samples. The Nigerian TTSuV1a and TTSuV1b shared a sequence identity of 91–100% and 95–100%, respectively, among each other. The ASFV sequences were 100% identical to members of genotype 1. This is the first report on the presence of TTSuV1a/b in domestic pigs in Nigeria and coinfection with ASFV. Although the prevalence of TTSuV1a/b in Nigeria was low, we recommend further studies to establish the trend and possible role in the pathogenesis of ASFV.

## 1. Introduction

Torque teno virus (TTV) is a small icosahedral and nonenveloped, single-stranded DNA (ssDNA) virus. It is circular with a negative genome that was first reported in a human with posttransfusion hepatitis in Japan [1]. The virus has also been reported to infect domestic animals such as pigs and boars [2, 3]. TTV are classified into the family Anelloviridae including 9 different genera among which is the genus* Iotatorquevirus.* Genetic analysis has shown that two genotypes of the genus* Iotatorquevirus* [torque teno sus virus 1 (TTSuV1) and torque teno sus virus 2 (TTSuV2)] and the newly grouped genotype TTSuVk2 of the genus* Kappatorquevirus* exist in pigs [4].

Torque teno viruses have been reported to be distributed globally with human TTV being ubiquitous while several other reports of swine TTSuV infection have been reported in Spain, Italy, Russia, China, and very recently Uganda in Africa [5–8]. TTSuV has been reported in coinfection with other pathogens but its evidence as a pathogen of pigs and its involvement in causality is yet to be elucidated [6].

The disease caused by TTSuV has not yet been defined even though it is widely spread and species specific. However, TTSuV2 (now TTSuV1b) has been reported in domestic reared pigs with other pathogens such as porcine circovirus 2 (PCV-2), hepatitis E virus (HEV), postweaning multisystemic wasting syndrome (PMWS), porcine endogenous retrovirus, and Ndumu virus [9–13]. On the other hand, TTSuV1a has been suggested to trigger PMWS development in gnotobiotic pigs coinfected with PCV-2 [14]. Furthermore, coinfection of TTSuV1a and* porcine reproductive and respiratory syndrome virus *(PRRSV) [15] has also been reported; Blomström et al. [12] have also reported a novel variant of porcine parvovirus 4 (PPV4) from bushpig (*Potamochoerus larvatus*) coinfecting with TTSuV1a and TTSuV1b. Therefore, the potential association of swine TTSuV with other disease occurrence in pigs is of scientific interest.

The rising demand for livestock products in Nigeria has resulted in government agricultural intervention leading to increased pig production [16]. However, with the advent of African swine fever in 1997, the prospect of the pig industry has continued to dwindle [17, 18]. Some regions in Nigeria preferred pig to other food animals due to its relative rapid growth rate, short cycle, and large litter size. Given the extensive/semi-intensive farming system common in Nigeria and the rising contact between pigs and humans in addition to poor farm practices, any potential risk from TTSuV or coinfection with other pathogens could lead to public health consequences in terms of the pig's capacity to serve as reservoir and/or transmitter of several emerging and reemerging diseases. As part of ASF surveillance in Nigeria, blood samples from selected slaughterhouses were analyzed to determine the presence of TTSuV1. The main objective of this study was to determine the presence of swine TTSuV1a and TTSuV1b genotypes in association with ASFVs in Nigeria.

## 2. Materials and Methods

### 2.1. Study Area and Sample Collection

**Figure 1:**
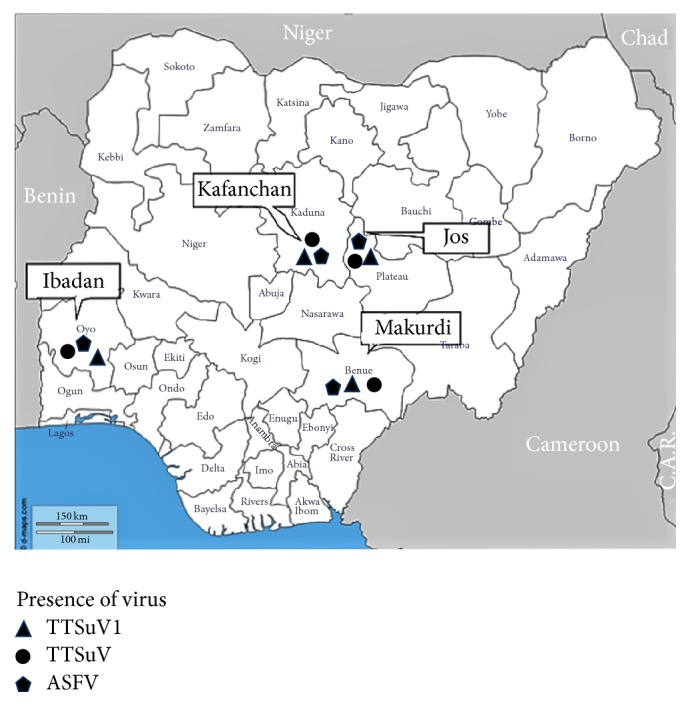
Map of Nigeria showing the 4 locations of slaughterhouses where samples were collected.

Blood samples from 181 domestic pigs were collected from four slaughterhouses from four localities in Nigeria: Jos, Makurdi and Ibadan abattoirs, and Kafanchan pig market slaughter slab (Figure 1). Sampling was carried out between January and March 2014 as part of a research project on the epidemiology of African swine fever in some pig producing states of Nigeria. This project was with the mandate of National Veterinary Research Institute (NVRI), Vom, and the Federal Department of Veterinary and Pest Control Services of the Federal Ministry of Agriculture, Abuja. Apparently healthy pigs presented to the slaughterhouses were sampled.

### 2.2. Sample Processing and DNA Extraction

Blood samples (*n* = 181) were collected in sterile sample bottles with ethylenediaminetetraacetic acid (EDTA) anticoagulant and kept at +4°C to +8°C until used for DNA extraction. The DNA extraction was carried out using a DNeasy blood and tissue kit (Qiagen, Hilden Germany) following the manufacturer's guidelines. Extracted DNA was kept at −20°C pending PCR.

### 2.3. Confirmation of ASFV by PCR

ASFV was confirmed using the primer pair ASF-1 and ASF-2 according to the Manual of Diagnostic Tests and Vaccines [19]. ASF specific primers targeting the major capsid protein (VP72 gene) 278-bp fragment within the conserved region were amplified as described by the OIE manual. A 478-bp C-terminus of the p72 gene was also amplified for genotyping as described by Bastos et al. [20].

### 2.4. TTSuV1 and TTSuV2 Detection and Partial Sequencing

The sample extracted DNA was used for the detection of TTSuV1a and TTSuV1b. Assessment of TTSuV genotypes 1a and 1b from the collected samples was analyzed by amplifying an untranslated region (UTR) of the TTSuV1 viral genome using species specific primers as reported by Segalés et al. [5]. The amplification was performed on a GeneAmp® PCR System 9700 machine (Applied BioSystems, USA).

The PCR amplicons were resolved on 1.8% agarose in Tris-borate-EDTA- (TBE-) buffered gels stained with ethidium bromide. Ten microlitres of the PCR product from each of the tubes was mixed with 1 *μ*L of 6x buffer and electrophoresed along with a 50-bp DNA molecular weight marker (GeneRuler, MBI Fermentas) at a constant voltage of 100 V for 45 min in 1x TBE buffer. Amplified products were viewed using a Bio-Rad Gel Doc™ XR system. The PCR positive products were purified using Wizard® SV Gel and PCR clean-up system (Promega Corporation, Madison, WI, USA) according to the manufacturers' instructions and eluted in 30 *μ*L EB. The purified products were sequenced by Inqaba Biotec®, South Africa.

### 2.5. Statistical Analyses of TTSuV Genotype Prevalence

The prevalence data generated with the screening of TTSuV and ASFV DNA in apparently healthy population was analyzed with statistical tests carried out in SPSS, version 17.0. Student's *t*-test, Pearson's correlation, and chi-square/Fisher's exact test were applied wherever relevant. *P* value <0.05 was considered significant.

### 2.6. Phylogenetic Analysis

The chromatograms were edited in SeqMan (Lasergene 9, DNASTAR Inc., Madison, USA). The edited sequences were subsequently aligned by ClustalW in BioEdit http://www.mbio.ncsu.edu/bioedit/bioedit.html. The phylogenetic relationship among the TTSuV1a and TTSuV1b sequences from this study was compared to previously published sequences available from GenBank http://www.ncbi.nlm.nih.gov/genbank using Mega 6.0 [21] for the construction of a phylogenetic tree using the Maximum-Likelihood algorithm with the Tamura 3-parameter model substitution with a bootstrap value of 1000.

## 3. Results

### 3.1. Detection of ASFV from Blood

Of the 181 samples collected from the four slaughterhouses, overall blood positivity rate for the pig populations was 12.71% (23/181). Location-wise, 77, 24, 42, and 38 blood samples were collected from Jos, Kafanchan, Ibadan, and Makurdi, respectively. A total of 9 (11.69%), 7 (29.17%), 5 (11.91%), and 2 (5.26%) were positive for ASFV from Jos, Kafanchan, Ibadan, and Makurdi, respectively (Table 1). Our result showed that Kafanchan had the highest number of pigs positive for ASFV and Makurdi was with the lowest number of ASFV positives.

### 3.2. Detection of TTSuV Genotypes from Blood

A total of 181 suspected samples collected from four slaughterhouses, Jos, Makurdi, Ibadan, and Kafanchan, were screened for ASFV and TTSuV1 genome, respectively. Of these samples 32 were positive for TTSuV1 genotypes 1a and 1b by TTSuV1F/R and TTSuV2F/R primer amplification from all the slaughterhouses. TTSuV1a and TTSuV1b infections were found in all the slaughterhouses in the four cities (Table 1). There was no statistically significant difference (*p* = 0.075) between the prevalence of genotype 1a infection alone (10.5%, 19/181) and 1b alone (7.2%, 13/181) for the four different locations. Nevertheless, there was a statistically significant (*p* = 0.011) difference between the genotypes with coinfection (1.7%, 3/181) and also between the overall TTSuV1 infections (17.7%, 32/181) with coinfection (*p* = 0.005) with ASFV (7.7%, 14/181). No coinfection of the genotype (TTSuV1a/TTSuV1b) was detected in Jos and Ibadan. Similarly, coinfection of TTSuV1/ASFV was detected in all the slaughterhouses with the exception of Makurdi (Table 1). TTSuV1a was more prevalent than TTSuV1b (Table 1).

### 3.3. Phylogenetic Relationships between TTSuV1 Genotypes

A total of 8 TTSuV1 sequences generated from PCR products and those from the GenBank were used to infer phylogeny. All the sequences obtained from this study were deposited in the GenBank with accession number KT160265-72.

**Figure 2:**
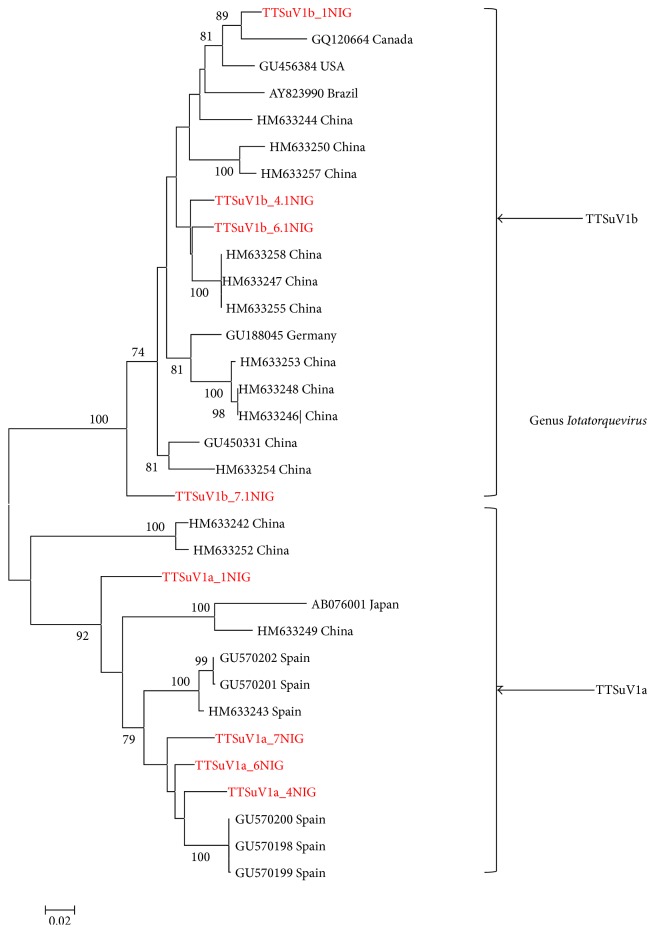
Molecular phylogenetic analysis of TTSuV1a and TTSuV1b sequences of the UTR region using Maximum-Likelihood method based on the Tamura 3-parameter model. A discrete gamma distribution was used to model evolutionary rate differences among sites (5 categories (+*G*, parameter = 0.3029)). Only bootstrap values above 70% are shown and sequences from this study are bold and highlighted (red).

The TTSuV1a sequences from our study revealed a sequence similarity of 91–100% to one another and 91%–97% to other sequences from the GenBank. The phylogenetic analysis of Nigerian TTSuV1a revealed no unique clustering with other global sequences from the GenBank (Figure 2).

Equally the Nigerian TTSuV1b sequences displayed a similarity of 95–100% among each other and 85%–97% when compared with 16 sequences from the GenBank. However, no clear geographical grouping was observed phylogenetically (Figure 2).

## 4. Discussion

To the best of our knowledge, this is the first study in Nigeria to investigate the presence of TTSuV1 in domestic pigs. Our present study confirms that the infection is prevalent (17.7%) in Nigerian domestic pigs infected with either of the 2 TTSuV1 genotypes. It is not clear whether sample number or sampling season played a role in the prevalence rate but our finding is in agreement with Brink et al. [8] in Uganda who reported 51.6% positivity from 95 samples collected during ASF studies. TTSuV1 was detected in all the 4 slaughterhouses but the prevalence was low compared to reports from China and Uganda [7, 8].

Interestingly the genotype TTSuV1b was found in fewer pigs when compared to TTSuV1a and only a few pigs (3/181, 1.7%) were coinfected with both genotype. This is in agreement with previous reports by Brinks et al. [8] in Uganda and Mei et al. [7] in China.

In addition, we also observed coinfection of TTSuV1 with ASFV in domestic pigs in Nigeria. Interestingly, TTSuV1 and ASFV coinfection was higher than TTSuV1a/b but overall coinfection with either of the genotypes (32/181, 17.7%) was higher than with ASFV (14/181, 7.7%). From the total number of samples analyzed, ASFV detection was 11.6%. ASFV has been reported to be endemic in Nigeria [17]; however the potential role of TTSuV1 in the infection and epidemiology of ASFV still needs to be explored. TTSuV1 coinfection has been reported with other diseases of pigs such as porcine circovirus associated diseases [14, 15]. In humans, TTV coinfection with other viruses has been described to enhance the pathogenic potential of the coinfecting viral agent and thereby worsen clinical manifestation [22–24].

Genetically, the similarity of Nigerian TTSuV1 UTR regions is higher for TTSuV1b (95–100%) than TTSuV1 (91–100%). Similarly, the sequence identity with other TTSuV1 globally revealed a related pattern (TTSuV1a: 91–97%; TTSuV1b: 85–97%). However, previous studies using the UTR region did not reveal distinct geographical clustering. The phylogenetic analysis of the Nigerian sequences showed TTSuV1a and TTSuV1b distribution among other sequences globally as described by ICVT (2011) new classification. However, sequences from our study revealed a similar pattern to TTSuV1 reports from Spain and Uganda [5, 8].

In contrast, other studies had suggested the use of ORF1 capsid gene as a marker for better resolution and molecular epidemiology because the gene is under selection pressure [5]. A complete genome sequencing has also been advocated for a better understanding of the virus dynamics [4, 25, 26].

ASFV sequences obtained from our study showed 100% sequence identity of the p72 gene to all other members of genotype 1 included in the analysis and phylogenetically clustered together (data not shown).

The capacity of TTSuV1 on its own to cause disease or influence ASF is yet to be fully elucidated, but it has been reported to be involved directly or indirectly in the development of other diseases such as PMWS and PCV-2. Its association with PCV-2 has been reported especially in diseased animals [12, 14]. From the total number of samples analyzed, 14 were positive for ASFV from apparently healthy pigs suggesting state of nonclinical manifestation of disease. Phylogenetically, the viruses were similar to those previously reported to be circulating in Nigeria. It is however possible that the pigs positive for ASFV brought to the slaughterhouse could be ASFV carriers without developing the disease or could have been brought into the slaughterhouse prior to visible signs or part of emergency sale.

## 5. Conclusion

We reported the circulation of TTSuV1 in Nigeria coinfecting with ASFV and like other parts of the world TTSuV1a/b infection is not very common among domestic pigs in Nigeria. However, having detected TTSuV1 coinfecting with ASFV and given the lethal nature of ASFV, we hypothesize that TTSuV1 may play an exacerbating role in the pathogenesis of ASF in domestic pigs in Nigeria. Further investigation needs to be carried out to establish if the trend is rising. The role of TTSuV in the infection and epidemiology of ASFV needs to be explored.

## Figures and Tables

**Table 1:** Prevalence of swine TTSuV1a and TTSuV1b species in some pig slaughterhouses in Nigeria.

Location	TTSuV1a	TTSuV1b	Overall	Coinfected TTSuV1a/TTSuV1b	Coinfected TTSuV1/ASF
Jos	12/77 (15.6%)	2/77 (2.6%)	14/77 (18.2%)	0/77 (0%)	4/77 (5.2%)
Kafanchan	3/24 (12.5%)	5/24 (20.8%)	8/24 (33.3%)	2/24 (8.3%)	6/24 (25.0%)
Makurdi	1/38 (2.6%)	2/38 (5.3%)	3/38 (7.9%)	1/38 (2.6%)	0/38 (0%)
Ibadan	3/42 (7.1%)	4/42 (9.5%)	7/42 (16.7%)	0/42 (0%)	4/42 (9.5%)
**Total positives**	**19/181 (10.5%)**	**13/181 (7.2%)**	**32/181 (17.7%)**	**3/181 (1.7%)**	**14/181 (7.7%)**
